# *Aeromonas* Diversity and Antimicrobial Susceptibility in Freshwater—An Attempt to Set Generic Epidemiological Cut-Off Values

**DOI:** 10.3389/fmicb.2017.00503

**Published:** 2017-03-28

**Authors:** Sandrine Baron, Sophie A. Granier, Emeline Larvor, Eric Jouy, Maelan Cineux, Amandine Wilhelm, Benoit Gassilloud, Sophie Le Bouquin, Isabelle Kempf, Claire Chauvin

**Affiliations:** ^1^Mycoplasmology-Bacteriology Unit, Ploufragan-Plouzané Laboratory, French Agency for Food, Environmental and Occupational Health and Safety (Anses)Ploufragan, France; ^2^Bretagne-Loire UniversityRennes, France; ^3^Laboratory for Food Safety, French Agency for Food, Environmental and Occupational Health and Safety (Anses), Paris-Est UniversityMaisons-Alfort, France; ^4^Laboratory for Hydrology, French Agency for Food, Environmental and Occupational Health and Safety (Anses)Nancy, France; ^5^Epidemiology and Welfare in Poultry and Rabbit Farming, Ploufragan-Plouzané Laboratory, French Agency for Food, Environmental and Occupational Health and Safety (Anses)Ploufragan, France; ^6^Epidemiology and Welfare in Pigs, Ploufragan-Plouzané Laboratory, French Agency for Food, Environmental and Occupational Health and Safety (Anses)Ploufragan, France

**Keywords:** *Aeromonas* spp., epidemiological cut-off, freshwater, ECOFFinder, normalized resistance interpretation method, antimicrobial resistance, minimum inhibitory concentration

## Abstract

The importance of the role of environment in the dissemination of antimicrobial resistant bacteria is now well recognized. Thus, bacterial indicators to monitor the phenomena are required. The *Aeromonas* genus is autochthonous in the aquatic environment and easy to detect in any water type, such as freshwater, or wastewater. These microorganisms are also causing infections in humans and animals (including fish). Furthermore, as *Aeromonas* spp. is able to acquire antimicrobial resistance mechanisms, it is candidate for indicator bacteria to follow antimicrobial resistance dissemination in aquatic environments. Unfortunately, to date, interpretation criteria for *Aeromonas* spp. for antimicrobial susceptibility tests are scarce in the literature. No epidemiological cut-off values for *Aeromonas* are currently available at EUCAST to interpret Minimum Inhibitory Concentrations (MIC). The only interpretation criteria available are clinical breakpoints from CLSI that are adapted from *Enterobacteriaceae*. Based on the results of MIC distributions obtained for a collection of environmental isolates of *Aeromonas*, this study aimed at proposing tentative epidemiological cut-off values (CO_WT_) for *Aeromonas* spp. assessing whether the genus is an acceptable level of definition. Thus, 233 isolates collected from 16 rivers were identified at species level using Maldi-Tof (Bruker). Eleven different species were identified, the most abundant were *A. bestiarum* (*n* = 54), *A. salmonicida* (*n* = 45), *A. sobria* (*n* = 41), and *A. eucrenophila* (*n* = 37). 96-well micro-plates containing different concentrations of 15 antimicrobials, namely cefotaxime, ceftazidime, chloramphenicol, colistin, enrofloxacin, erythromycin, florfenicol, flumequine, gentamicin, nalidixic acid, oxolinic acid, streptomycin, temocillin, tetracycline, and trimethoprim-sulfamethoxazole, were prepared. The broth micro-dilution method was used to determine the antimicrobial susceptibility of each isolate. The estimation of CO_WT_ values was satisfactory obtained at genus level for all antimicrobials except cefotaxime and erythromycin. This first step is an invitation for other research teams to increase the amount of antimicrobial resistance data collected. Then, robustness of our proposed provisional generic epidemiological cut-off values could be assessed by testing antimicrobial susceptibility of various *Aeromonas* collections.

## Introduction

Antimicrobial agents have revolutionized medicine in many respects, but their use has been accompanied by a rapid emergence of resistant strains, resulting now in a global health issue. Shared use of antibiotics in both Humans and animals is a growing public health concern. Human and animal infectious diseases are so closely interlinked in a common environment that the One World - One Medicine - One Health concept fully applies to tackle the growing issue of antibiotic resistance. People and animals are connected to each other through the environment (including air, water, soil…). Aquatic environments may provide an ideal setting for acquisition and dissemination of antibiotic resistance: (i) they are frequently impacted by anthropogenic activities (wastewater, runoff, aquatic farms) (Marti et al., 2014), (ii) they contain an autochthonous bacterial microbiota which harbors antimicrobial resistance associated genes, (iii) they allow the mix of bacteria from different origins (human, livestock…) (Rizzo et al., 2013), and (iv) they may contain antimicrobials or biocides which may select resistant bacteria.

*Aeromonas* is an autochthonous bacteria of aquatic environment, which can be isolated from virtually any water source including freshwater (Goñi-Urriza et al., 2000), estuarine environments (Silva et al., 2014), drinking waters (Pablos et al., 2009), wastewaters and sewage (Imziln et al., 1996). This genus is a major causative agent of infections in fish (Austin, 2015), indeed an increasing range of *Aeromonas*, including *A. allosaccharophila, A. bestiarum, A. caviae, A. hydrophila, A. jandaei, A. salmonicida, A. schubertii, A. sobria biovar sobria*, and *A*. *veronii* biovar *sobria*, have become associated with disease of predominantly freshwater fish in most countries (Figueras and Baez-Higalgo, 2015). Among them, *A. hydrophila, A. caviae*, and *A. veronii* have been associated with human diarrheal diseases and wound infections (Janda and Abbott, 2010; Shin et al., 2015). Natural transformation is a general property of *Aeromonas* environmental isolates (Huddleston et al., 2013). Moreover, integrons, and other genetic elements are frequently detected in *Aeromonas*, in respect with these properties, *Aeromonas* spp. has been studied as an indicator of the dissemination of antimicrobial resistance in water (Usui et al., 2016; Varela et al., 2016) or in fish (Naviner et al., 2006, 2011) excepted for ampicillin, amoxicillin-clavulanate and cefazolin which is an intrinsic resistance for *Aeromonas* (CLSI, 2015). Monitoring *Aeromonas* susceptibilities would be much more relevant if standard interpretative criteria, internationally agreed, are applied to the generated data.

To study the antimicrobial susceptibility, clinicians and epidemiologists/ecologists/microbiologists have two totally different approaches, clinicians focus on the tryptic microorganism/antibiotic/host and others on the pair microorganism/antibiotic. Clinicians need to choose the right treatment in order to have the best chance to achieve the complete recovery of their patient and avoid development of antimicrobial resistance. In order to predict the outcome of the treatment, they need to use so called “Clinical breakpoints.” Clinical breakpoints allow them to interpret an *in vitro* measure or estimation of the minimum inhibitory concentration (MIC), to categorize their result as Susceptible/Intermediate/Resistant, meaning high likelihood of therapeutic success/uncertain therapeutic effect/ high likelihood of therapeutic failure.

Epidemiologists and microbiologists are mainly interested in evolution or emergence of bacterial populations displaying resistant traits, regardless of any therapeutic outcome. Epidemiological cut-off values allow them to interpret an *in vitro* measure or estimation of the MIC taking into account only the pair microorganism/antibiotic to categorize microorganisms as wild type or non-wild type, meaning for absence or presence of any acquired and mutational resistance mechanism to the drug in question. These interpretive criteria, called ECVs and ECOFFs by CLSI and EUCAST respectively, are based on data derived from diverse laboratories and represent the upper limit of the distribution of MIC data of fully susceptible (wild type) strains.

For *Aeromonas*, ECVs are available but only regarding the species *A. salmonicida* and for florfenicol, ormethoprim-sulfadimethoxine, oxytetracycline and oxolinic acid either MICs or for Inhibition Zone Diameter (IZD) obtained by disk diffusion and for gentamicin, erythromycin, and trimethroprim-sulfamethoxazole (only IZD) (VET03/VET04-S2) (CLSI, 2014b).

If antibiotic susceptibility of clinical isolates of *Aeromonas* has been extensively studied, less is known about environmental strains and particularly those from freshwater not directly impacted by wastewater input.

The aim of our study was to determine MICs of 15 antimicrobial agents for a collection of *Aeromonas* isolates from freshwater autochthonous flora. From these data, we propose a first set of presumptive interpretative criteria called CO_WT_ (Smith et al., 2016) for *Aeromonas* spp.

## Materials and methods

### Bacterial isolates

During 2014, 16 rivers located in the west part of France were sampled. Fourteen rivers were sampled once in winter (February/March) and once in summer (June/July) and two were sampled thrice in winter and thrice in summer. Each water sample was duplicated, and three volumes of each were analyzed. 10 and 1 mL were filtered onto 0.45 μm cellulose ester membranes (Millipore, Watford, UK), then filters were transferred onto glutamate starch phenol-red agar (GSP–Merck) and 0.1 mL was streaked onto GSP agar. The petri dishes were incubated at 22 ± 1°C for 48 h. Yellow colonies on GSP were considered as presumptive *Aeromonas*. Ten colonies per sample were purified on CHROMagar™ Orientation. The identification of isolates was confirmed at the genus level by PCR (Khan et al., 2009) and identification at the species level was done by Maldi-Tof (Microflex®Bruker V4.0.0.1_4613-5627). Up to three *Aeromonas* isolates per water sample were included in this study and stored at −20°C in peptone water with 20% glycerol.

### Determination of MICs

The broth micro-dilution method (CLSI, 2006) (VET04-A) was used to determine the MICs of 15 antimicrobial agents for *Aeromonas* isolates. A stock solution of each antimicrobial agent at 200X concentration was prepared with the solvent recommended by CLSI (2016) (VET04-A) and aliquots were stored at −70°C. Solutions of antimicrobial were diluted 1:100 on the day of testing. 96-well microplates (tissue culture plate, 96 well flat bottom with low evaporation led, Corning) were used. One hundred microliters of the antimicrobial solution were added to the first column of the microplate, and 50 μL of sterile water were added into each well of the microplate (excepted those of the first column). Serial two-fold dilutions of antimicrobial solution were performed by transferring 50 μL from column 1 to column 2 and so on up to column 12, to obtain final concentrations of cefotaxime (0.031–64 mg/L), ceftazidime (0.031–64 mg/L), chloramphenicol (0.062–64 mg/L), colistin (0.025–51.2 mg/L), enrofloxacin (0.008–16 mg/L), erythromycin (0.031–64 mg/L), florfenicol (0.062–128 mg/L), flumequine (0.008–16 mg/L), gentamicin (0.062–64 mg/L), nalidixic acid (0.062–128 mg/L), oxolinic acid (0.008–16 mg/L), streptomycin (0.125–256 mg/L), temocillin (0.125–256 mg/L), tetracycline (0.062–128 mg/L) and trimethoprim-sulfamethoxazole (0.031/0.589–8/152 mg/L). In this study, the five antimicrobial agents labeled in French aquaculture (flumequine, oxolinic acid, trimethoprim-sulfamethoxazole, tetracycline, and florfenicol), and other main antimicrobial agents usually used to monitor the antimicrobial resistance of gram negative bacteria were tested. In addition, temocillin was used to evidence carbapenemase production (Woodford et al., 2014).

The day before the MIC determination assay, colonies of *Aeromonas* were inoculated onto Mueller Hinton (MH) agar and incubated at 22 ± 1°C for 24 h. 0.5 McFarland bacterial suspensions, prepared in physiological water, were diluted 1:100 in cation-adjusted MH broth, in order to reach the final concentration of 5 × 10^5^ CFU/mL. Fifty microliters of the suspension were added to each well of the microplate. Two wells were used as positive controls (wells with only bacterial suspension) and two as negative controls (wells with only sterile cation-adjusted MH broth used to prepare the inoculum). The microplates were incubated at 22 ± 1 C for 24 h ± 2 h.

*Escherichia coli* ATCC 25922 and *A. salmonicida subsp salmonicida* ATCC 33658 were used as controls, and incubated respectively at 35 ± 1°C and 22 ± 1°C. For all the isolates of *Aeromonas* tested, the density of the inoculum was controlled by inoculation of MH agar with 10 μL of the suspension from the positive control well before incubation of the microplate.

### MIC analysis and provisional epidemiological cut-off values (CO_WT_) determination

From the distribution of MICs values obtained, MIC_50_, MIC_90_, and CO_WT_ were calculated. The abbreviation CO_WT_ will be used to refer to these results as the values are proposals based on this isolate collection. The abbreviations ECV and ECOFF will not be used as they refer to consensus-based epidemiological cut-off values from CLSI and EUCAST, respectively.

Provisional CO_WT_ values were statistically determined according to two methods, one proposed by Turnidge et al. and second one by Kronvall (Turnidge et al., 2006; Kronvall, 2010) which will be referred to later on as “Turnidge method” or “Kronvall method.” Fully automated and freely available Excel spreadsheet calculators to apply the normalized resistance interpretation (NRI) method (Kronvall, 2010) [available at http://www.bioscand.se/nri/ used with permission from the patent holder, Bioscand AB, TÄBY, Sweden (European patent No 1383913, US Patent No. 7,465,559)] and ECOFFinder MS (available at http://clsi.org/standards/micro/ecoffinder/) were used. Following their author's recommendation, CO_WT_ were computed for 97.7 and 99% of the population level inclusion in the wild type population, respectively. Numbers and percentages of non-wild type isolates were calculated afterwards.

Calculations were performed for each antimicrobial, at genus level on the whole dataset and at species level, when at least 30 isolates from the same species were encountered in the collection (CLSI Report cited by Smith et al., 2013).

## Results

### Species diversity

**Figure 1:**
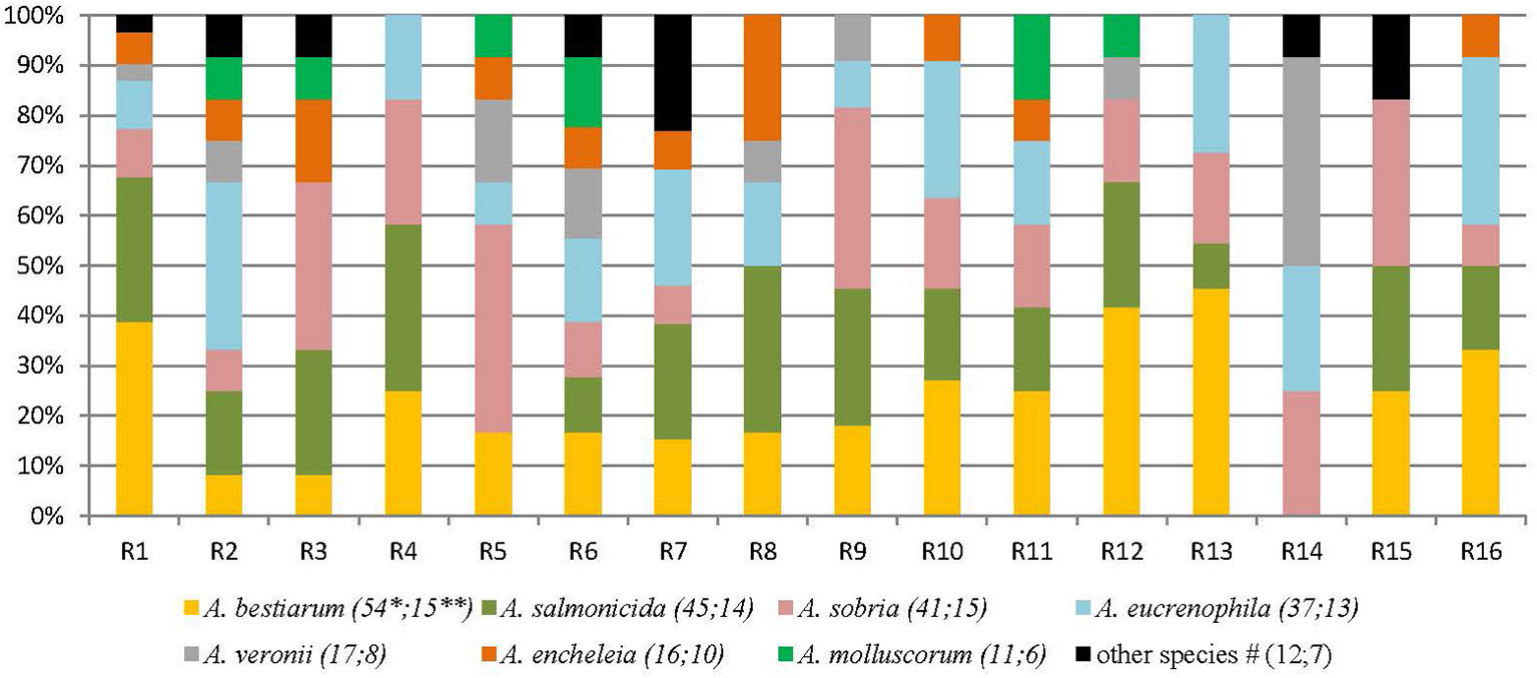
**Diversity of ***Aeromonas*** species isolated from different freshwater sampling points (France, 16 rivers)**. R: river. ^*^Number of isolates of this species. ^**^Number of rivers where the species was detected at least one time. ^#^Other species correspond to: 4 *A. popoffii*, 3 *A. caviae*, 2: *A. media*, 2 *A*. spp and 1 *A. hydrophila*; and were detected as follow: R1 1 *A. popoffii*; R2 1 *A*. spp; R3 1 *A. media*; R6 1 *A*. spp and 2 *A. caviae*; R7 1 *A. popoffii*, 1 *A. hydrophila*, and 1 *A. caviae*; R14: 1 *A. media*; R15: 2 *A. popoffii*.

233 isolates of *Aeromonas* spp. were collected from the 16 rivers (Figure 1). *Aeromonas* spp. was detected in all the 16 sampled rivers, in winter and in summer. In four rivers, less than six isolates were included due to the non-confirmation of the presumptive identification *Aeromonas* (data not shown). Eleven species were detected and two isolates could not be identified at the species level. *A. bestiarum* (*n* = 54; 23.1%), *A. salmonicida* (*n* = 45; 19.3%), *A. sobria*, (*n* = 41; 17.6%), *A. eucrenophila* (*n* = 37; 15.9%), and *A. veronii* (*n* = 17; 7.3%) were the five most abundant species and they accounted for 83.3% of the 233 isolates of *Aeromonas* included in this study. The frequency of species isolation did not differ between summer and winter (Wilcoxon test *p* = 0.9). Pooling winter and summer samples from the same river together, at least four different species per river were detected.

### Antimicrobial susceptibility

For each MIC determination assay, the results obtained for the reference strains and the density of the inoculum complied with CLSI recommendations (data not shown) (CLSI, 2006). Distribution of the MICs of 15 antimicrobials and the corresponding MIC_50_ and MIC_90_ are displayed in Table 1. MIC values below the tested ranges were observed for 12 out of the 15 antimicrobial agents. For six of them (florfenicol, trimethoprim-sulfamethoxazole, chloramphenicol, ceftazidime, streptomycin, and temocillin), less than 5% of the MIC values were concerned. The most important proportions of isolates displaying MIC below the tested range were observed for the quinolone class (oxolinic acid (*n* = 87; 37.3%), nalidixic acid (*n* = 75; 32.3%), enrofloxacin (*n* = 39; 16.7%) and flumequine (*n* = 12, 5.1%), followed by cefotaxime (*n* = 48; 20.6%) and tetracycline (*n* = 15; 6.4%).

**Table 1:**
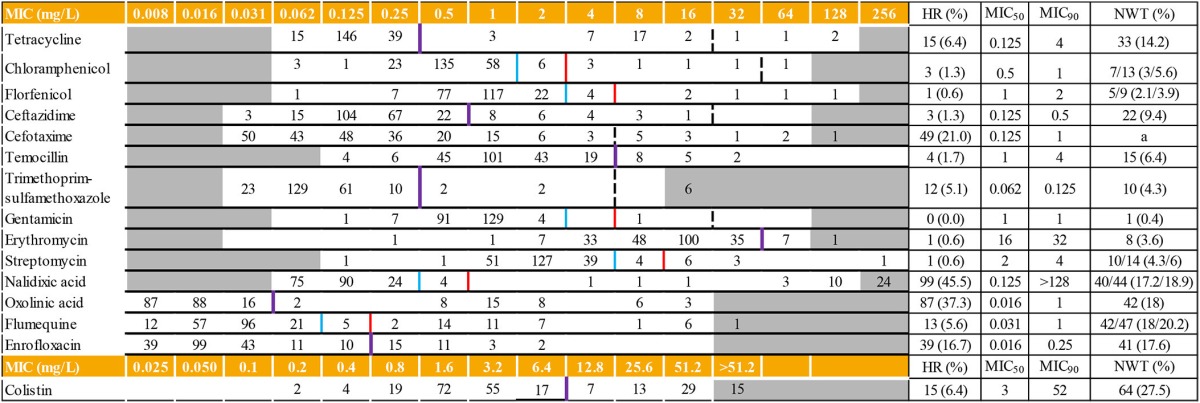
**Distribution of MICs (mg/L) in 233 isolates of ***Aeromonas*** spp. and interpretive criteria**.

*White fields represent the range of dilutions tested. MIC values equal to or lower than the lowest concentration tested are presented as the lowest concentration. MIC values greater than the highest concentration tested are presented as one dilution step above the test range*.

*Red, blue and dotted vertical lines indicate CO_WT_ calculated applying Kronvall method, CO_WT_ calculated with Turnidge method (in case of agreement purple lines are therefore represented) and clinical breakpoint proposed by CLSI, respectively for Aeromonas spp. No CO_WT_ was calculated for cefotaxime as the mode was out of the range*.

*HR (%): number (percentage) of isolates for which the MIC value was not in the range test, ie value below and above the range. So in several cases, number of isolates in the raw of the lower concentration included isolates with MIC below the range*.

*NWT (%): number (percentage) of isolates of non-wild type using CO_WT_values resulting from Kronvall/Turnidge method respectively*.

*a: no CO_WT_ was calculated*.

On the opposite, MIC values above the tested ranges were observed for 44 isolates and six antimicrobial agents: cefotaxime, erythromycin, trimethoprim-sulfamethoxazole, nalidixic acid, flumequine, and colistin. Except for cefotaxime, the mode was always included in the range.

For gentamicin, the MIC_50_ and MIC_90_ values were the same: 1 mg/L. For seven out of the 15 tested antimicrobials (tetracycline, cefotaxime, nalidixic acid, oxolinic acid, flumequine, enrofloxacin and colistin), differences between MIC_50_ and MIC _90_ were at least three dilutions.

MIC_50_ values calculated for *Aeromonas* spp. (${\text{MIC}}_{50}^{\text{genus}}$) (*n* = 233 isolates; 11 different species) were the same, within one dilution step, as those calculated for the four most abundant species (Table 2). Cefotaxime was the unique exception: MIC_50_ of the 41 isolates of *A. sobria* was smaller than the ${\text{MIC}}_{50}^{\text{genus}}$, 0.031 mg/L, and 0.125 mg/L, respectively.

**Table 2:** **CO_**WT**_, MIC_**50**_, and MIC_**90**_ (mg/L) values specific for ***A. bestiarum, A. salmonicida, A. sobria***, and ***A. eucrenophila*** and CO_**WT**_ for ***Aeromonas spp*****.

**Antimicrobial agent**	***A. bestiarum*** **(*****n*** = **54)**			***A. salmonicida*** **(*****n*** = **45)**			***A. sobria*** **(*****n*** = **41)**			***A. eucrenophila*** **(*****n*** = **37)**			***Aeromonas spp*. (*n* = 233)**
	**MIC_50_**	**MIC_90_**	**CO_WT_ T/K**	**MIC_50_**	**MIC_90_**	**CO_WT_ T/K**	**MIC_50_**	**MIC_90_**	**CO_WT_ T/K**	**MIC_50_**	**MIC_90_**	**CO_WT_ T/K**	**CO_WT_ T/K**
Tetracycline	0.125	0.25	0.25^*^	0.125	0.25	0.25	0.25	8	0.25	0.125	0.25	0.25/0.5	0.25
Chloramphenicol	0.5	0.5	NC/1	0.5	1	1/2	0.5	0.5	1/2	0.5	1	1/2	1/2
Florfenicol	1	1	2	1	1	2	0.5	1	1	1	2	2	2/4
Ceftazidime	0.25	0.5	0.5	0.125	0.5	0.5	0.125	0.25	0.25/0.5	0.125	1	0.25/NC	0.5
Cefotaxime	0.125	0.5	NC	0.125	2	NC	<0.031	0.125	NC	0.125	1	NC/0.5	NC
Temocillin	1	2	2/4	1	2	2	0.5	2	8/4	1	2	4	4
Trimethoprim-sulfamethoxazole	0.062	0.125	0.125/0.25	0.062	0.125	0.125/0.25	0.125	2	0.25/0.5	0.062	0.25	0.125/0.25	0.25
Gentamicin	0.5	1	1/2	1	1	NC/2	1	1	2	1	1	NC/2	2/4
Erythromycin	16	32	64/8	16	32	NC	8	8	16/32	16	32	64/16	32
Streptomycin	2	2	NC/8	2	2	4	4	16	8	2	4	4/8	4/8
Nalidixic acid	0.125	0.25	0.125/0.25	0.125	0.125	0.25	0.125	>128	0.125	0.125	4	0.25	0.25/.5
Oxolinic acid	0.016	0.031	0.031	0.016	0.016	0.031	0.016	1	0.016	0.016	0.5	0.031	0.031
Flumequine	0.031	0.031	0.062/0.125	0.031	0.062	0.062	0.031	1	0.062/0.031	0.031	0.25	0.062	0.062/0.125
Enrofloxacin	0.016	0.031	0.031	0.016	0.031	0.031/0.062	0.031	0.5	0.016/0.031	0.016	0.125	0.062	0.125
Colistin	3.2	51	12.75/25.5	6.4	51	12.75/NC	1.6	3.2	6.4	3.2	51	6.4	6.4

*T/K, Turnidge and Kronvall methods respectively. NC, CO_WT_ could not be computed*.

* *A unique value means that the CO_WT_ values are identical for both methods*.

For the phenicols class (chloramphenicol and florfenicol), gentamicin and temocillin, the ${\text{MIC}}_{90}^{\text{genus}}$ and MIC_90_ of the four most abundant species values were not significantly different, with a maximum of one dilution step variation.

CO_WT_ were calculated for 14 antimicrobial agents for the complete *Aeromonas* spp. dataset (Table 1). For all tested antimicrobials, MIC standard deviation never exceeded 1.2 log_2_ μg/ml, the limit value implemented in Kronvall method spreadsheet (Kronvall, 2010). No value was computed for cefotaxime due to the truncated distribution of isolates with a high number of strains out of the tested dilution range (Table 1). Similar CO_WT_ values were obtained by Kronvall and Turnidge methods applied for eight antimicrobials (61.5%). For the cases in which values were different, values obtained by the Turnidge method were consistently lower than the Kronvall method result by one dilution step. Percentages of non-wild type strains ranged from 0.4 to 27.5%, colistin, quinolone compounds, and tetracycline displaying the highest percentages. Excluding cefotaxime, 107 strains (45.9%) could be considered as wild type (WT) for all tested antimicrobials. The second most frequent phenotype, reduced susceptibility to colistin only, was represented by 47 strains (20.2%). Remaining isolates were distributed among 46 different combinations of non-wild type (NWT) and WT to the different antimicrobials (1 to 10 isolates per category).

Tentative species-specific CO_WT_ values (Table 2) could be computed by at least one statistical approach for all combinations excepted for cefotaxime. The values obtained by Turnidge or Kronvall methods were within one dilution step for a given pair antimicrobial-*Aeromonas* species, except for erythromycin. In the frame of one of the two methods, *Aeromonas* spp. and species-specific CO_WT_ were similar or within one dilution step for most of the species-antimicrobial combinations. Finally, combining all computed values obtained from both methods, for each antimicrobial tested except erythromycin, CO_WT_ ranges are at most three dilution step wide.

## Discussion

The advantage of reporting MIC distributions is to allow comparison of studies over a long period of time even if interpretative criteria change over time (Schwarz et al., 2010), provided that the MIC determination methods are comparable. The ability to follow antimicrobial susceptibility trend over a long period of time is crucial to monitor antimicrobial resistance dissemination in the environment.

MIC distributions appeared to be bimodal for some agents: two clearly distinct populations were identified for oxolinic acid, tetracycline, and colistin. From these distributions, calculated MIC_50_ and MIC_90_ values were compared to previously published ones, even though laboratory methods were slightly different. Gentamicin MIC_50_ and MIC_90_, calculated in this study (1 mg/L), were equivalent to those obtained by Kämpfer et al. (1999) on a collection of 217 *Aeromonas* genomic species from various origins (1 and 2 mg/L) (Kämpfer et al., 1999), by Goñi-Urriza et al. (2000), on a collection of 138 *Aeromonas* spp. isolated from freshwater (1 and 2 mg/L) and by Lamy et al. (2012) on a collection of 146 isolates from clinical and environmental origins (0.5 and 1 mg/L) (Kämpfer et al., 1999; Goñi-Urriza et al., 2000; Lamy et al., 2012). For chloramphenicol, MIC_50_ and MIC_90_ were 0.5 mg/L and 1 mg/L, respectively; same as Kampfer et al. and very similar to Goñi et al. results, 1 mg/L and 2 mg/L, respectively. Here, MIC_50_ for trimethoprim-sulfamethoxazole (0.062/1.178 mg/L) were lower than those observed in the three latter studies (1/19 mg/L in Kämpfer et al., 0.25/4.75 mg/L for Lamy et al. and 8/152 mg/L for Goñi et al).

In their study, Goñi-Urriza et al. (2000), considered that *Aeromonas* spp. was poorly susceptible to streptomycin due to a MIC_50_ value of 16 mg/L; ${\text{MIC}}_{50}^{\text{genus}}$ here was lower (2 mg/L). A MIC_90_ value of 16 mg/L was observed for the 41 studied isolates of *A. sobria*. Isolates here seemed to be more susceptible to streptomycin. Similarly, MIC_50_ for tetracycline was lower in the present study (0.125 mg/L vs. 0.5 mg/L). For cefotaxime, MIC_50_ was very similar (<0.1 mg/L vs. 0.125 mg/L). For colistin, MIC_90_ here was higher with a value of 52 mg/L vs. 2 mg/L which could be linked to the species composition of the collection. The MIC_90_ values for *A. bestiarum, A. salmonicida*, and *A. eucrenophila* were 52 mg/L although MIC_90_ for *A. sobria* was 3.2 mg/L.

Two types of thresholds are available: “Clinical breakpoints” to estimate the odds of therapeutic success to treat infections and “epidemiological cut-off values” to recognize any emerging resistance mechanism in the bacterial population studied. To delineate WT from NWT *Aeromonas* isolates, epidemiological cut-off values are needed.

In document M45-3rd Edition from CLSI, clinical breakpoints for 19 antimicrobial agents are proposed for *Aeromonas* spp. (CLSI, 2015). *Aeromonas* spp. includes members of *Aeromonas caviae* complex, *Aeromonas hydrophila* complex, and *Aeromonas veronii* complex. A footnote in the document mentions that most of the published data on susceptibility testing are limited to these three *Aeromonas* complexes. Moreover, the interpretative criteria are adapted from those for *Enterobacteriaceae*. To the best of our knowledge, all published studies on environmental sourced *Aeromonas* susceptibility tests, whatever their origin was freshwater (Rhodes and Kator, 1994; Imziln, 2001), wastewater (Imziln et al., 1996; Igbinosa and Okoh, 2012; Khor et al., 2015; Kim et al., 2015), aquaculture plants (Penders and Stobberingh, 2008), drinking water (Figueira et al., 2011), used these breakpoints. So, information gathered in those studies might document the hazard represented by these isolates in the context of human infections, but does not fully enquire about the issue of environmental antimicrobial resistance dissemination. In this specific environmental study, focusing on dissemination of antimicrobial resistance in an ecosystem, we found more relevant to consider epidemiological cut-off values as we only focus on the pair microorganism/antibiotic and no host or treatment option is involved.

Numerous methods were proposed to determine CO_WT_, from “eye-ball” determination to statistically oriented ones (Turnidge et al., 2006; Turnidge and Paterson, 2007; Kronvall, 2010; Hombach et al., 2014; Jaspers et al., 2014). These methods were applied in the present study according to their authors' recommendation, computing CO_WT_ for 99 and 97.7% of the population level inclusion in the wild type population. Results from these methods were in accordance, with frequent full agreement or one dilution step difference. Values could also be computed using Jaspers method (Jaspers et al., 2014-*data not shown*) for three antimicrobials (colistin, erythromycin, and temocillin) and were in full agreement with those obtained with other methods. Erythromycin CO_WT_ here computed (32 mg/L) should be interpreted cautiously considering the fact that MICs of the supposed WT population are distributed over eight dilution steps instead of three to five usually.

Few epidemiological cut-off values for *Aeromonas* could be found in the literature. On The European Committee on Antimicrobial Susceptibility Testing website (www.eucast.org), MIC distributions are available for *Aeromonas* spp., but no ECOFFs have been proposed due to the low number of observations. In the frame of a simulation study to determine robustness of CO_WT_, Smith and Kronvall published computed values for *Aeromonas* spp. and *A. salmonicida*, for oxytetracycline, oxolinic acid and florfenicol (Smith and Kronvall, 2015). The same value of 2 mg/L was proposed for florfenicol by both studies. For oxolinic acid, the value computed by Smith and Kronvall (2015) was 0.06 mg/L for *Aeromonas* spp. which is in accordance with our value (0.031 mg/L) considering the double dilution agreed variation for broth micro-dilution method (Smith and Kronvall, 2015). For *A. salmonicida* the value computed by Smith and Kronvall on a different dataset was 0.125 mg/L which is similar to the interpretive value proposed by CLSI (2014a). The CLSI ECVs for *A. salmonicida* were established based on visual inspection of MIC distributions for 217 isolates (Miller and Reimschuessel, 2006). In addition to oxolinic acid CLSI interpretive values were published for florfenicol (4 mg/L) oxytetracycline and ormetoprim-sulfadimethoxine (CLSI, 2015).

CO_WT_ were estimated for *Aeromonas* spp. and for the four main species encountered in the collection, in order to check for any species dependency upon the results. At species level, the available number of isolates was below the CLSI recommendation of 100 isolates (Smith and Kronvall, 2015) but close to the recommended minimal number of 30 WT isolates to form a Gaussian distribution as mentioned by Smith et al. (2013) and confirmed by Smith and Kronvall (2015). Nevertheless, some of the MICs distributions did not allow CO_WT_ calculation and values should be considered cautiously and unprecise (Smith et al., 2013) due to the low isolate number within a species. Species level values were mainly equal to or in the range of one dilution step around the CO_WT_ values determined for *Aeromonas* spp. (except 3/104 values: *A. bestiarum* and erythromycin CO_WT_ computed by Kronvall method, *A. bestiarum* and colistin CO_WT_ computed by Kronvall method, *A. sobria*, and enrofloxacin CO_WT_ computed by Turnidge method). These results do not preclude usage of CO_WT_ determined at the genus rather than the species level. The definition of CO_WT_ at the genus level has previously been considered (Miller and Reimschuessel, 2006; Smith et al., 2013) and applied (Miller and Reimschuessel, 2006; Smith et al., 2012; Smith and Kronvall, 2015), but contravenes to the generic principles established to set-up CO_WT_ values (Kronvall, 2010). Smith et al. (2012) addressed the question of the validity of *Aeromonas* genus defined CO_WT_ for antibiotic disk diffusion data, through the exploration of standard deviation of calculated normalized distribution (Smith et al., 2012). We addressed the same question comparing CO_WT_ values obtained at the genus and specific level, on a limited number of *Aeromonas* species. As concluded by Smith et al. (2012) for some agents, our results provide no reason why a single set of interpretative values could not be defined for application to all *Aeromonas* species included in our study. By projecting into daily business of a routine laboratory, identification of the genus *Aeromonas* is easy and reliable, even phenotypic methods could be used (Lamy et al., 2010) and several PCR were described, whereas identification at the species level requires either sequencing or Maldi-Tof methods. Moreover this genus is abundant and detectable easily through selective media. Thus, establishing CO_WT_ at genus level when possible seems more relevant to be widely used in future epidemiological studies. As emphasized by Smith et al. (2013) the workload would be greatly reduced defining interpretive criteria at the genus level.

Values obtained in this study are putative ones and should not be considered as official interpretative criteria. Our values are based on one laboratory only whereas multiple and diverse sources and a large number of isolates are recommended to offer more precise estimates (Smith et al., 2013) to reassess relevant generic CO_WT_s.

Recently, *Aeromonas* spp. was proposed as a potential indicator of antimicrobial susceptibility for aquatic environment by several authors (Usui et al., 2016; Varela et al., 2016). Indeed, *Aeromonas* genus is ubiquitous and its abundance in aquatic environment allowed its detection all along the year. Identification methods of the *Aeromonas* genus are reliable and costless, which is not the case at species level (Lamy et al., 2010). Yet large environmental studies on such a complex matrix as water enforce the need of easy and cheap tools in order to analyze a large amount of samples. Indeed a large amount of samples is the only way to apprehend the complexity and the ecological condition variability of the water matrix. Harmonization of susceptibility tests at *Aeromonas* genus level would probably allow collecting multiple observations to follow antimicrobial resistance traits in the aquatic environment.

## Conclusion

Thus, as a first step, it appears to be relevant to determine *Aeromonas* spp. CO_WT_ values. Further experiments might allow refining these values.

If *Aeromonas* spp. is used as an indicator of antimicrobial susceptibility for aquatic environment, it is absolutely essential to set epidemiological cut-off values; but this is far from being enough to be able to share and compare data. Indeed, if methods for assessing fecal contamination of water are standardized, it is absolutely not the case for *Aeromonas* spp. detection. Harmonization of the methods for detection, identification and characterization of *Aeromonas* is urgently needed.

## Author contributions

SB, IK, EL, EJ, CC, SG, and SL contributed to the design of the study. SB, EL, MC, BG, AW, and CC produced data. All authors contributed to the analysis of the data, to the redaction and/or the edition of the article.

## Funding

This study was financed through “AQUARES,” research project supported by both French Ministries in charge of Environment and Agriculture. AQUARES is part of the national effort to reduce antimicrobial resistance in veterinary medicine called “EcoAntibio2017.”

### Conflict of interest statement

The authors declare that the research was conducted in the absence of any commercial or financial relationships that could be construed as a potential conflict of interest.
